# Diagnostic Challenges in the Detection of Actinomycotic Osteomyelitis of the Mandible: A Case Report

**DOI:** 10.1155/crid/6211159

**Published:** 2025-01-04

**Authors:** Elitsa G. Deliverska, Dimitar T. Yovchev, Marusia Zh. Genadieva, Sadeta J. Parusheva

**Affiliations:** ^1^Department of Dental, Oral and Maxillofacial Surgery, Faculty of Dental Medicine, Medical University, Sofia, Bulgaria; ^2^Department of Imaging and Oral Diagnostics, Faculty of Dental Medicine, Medical University, Sofia, Bulgaria; ^3^Department of Pathology, Aleksandrovska University Hospital, Medical University, Sofia, Bulgaria

**Keywords:** *Actinomyces*, *Actinomyce*s-associated lesions, actinomycotic osteomyelitis, CT, jaw infection, MRI

## Abstract

Actinomycosis is a rare chronic granulomatous infection and can be caused by Gram-positive anaerobic bacteria which are normal commensals of the oral cavity and pharynx. These organisms can involve different parts of the maxillofacial region, rarely affecting the jaws. Actinomycotic osteomyelitis is an infection of the jaw bones, typically associated with trauma or an underlying nonspecific infection or disease. Herein, we describe a rare case of actinomycotic osteomyelitis of the mandible in a healthy male patient. Comprehensive surgical treatment with curettage and peripheral ostectomy was performed. The diagnosis was made on the basis of clinical findings and the histological result. Antibiotic treatment from the penicillin group was prescribed for a period of 1 month. Diagnosis is often delayed and can be a challenge for clinical practice due to the lack of criteria other than definitive histopathological examination. A chronic clinical course without regional lymphadenopathy may be essential in the diagnosis. A number of diseases can present with similar symptoms, necessitating strict adherence to a correct surgical protocol; even when such an infection is suspected, histopathological examination is essential, and long-term treatment with penicillin is advised for accurate treatment of actinomycotic infection.

## 1. Introduction

Historically, Langenbach was the first researcher to document this disease in humans in 1848 [1, 2]. In 1891, Israel and Wolff have isolated an anaerobic filamentous organism in humans, which was later named *Actinomyces israelii* in 1898 [1–3]. *A. israelii* is a Gram-positive microorganism, not fixed by acid, and produces sulfur granules. *Actinomycetes* strains resemble both bacteria and fungi; thus, they are often considered transitional between the two groups of microorganisms [2, 3]. They are anaerobic or facultative anaerobes unlike pathogenic fungi which are aerobic. These microorganisms can be isolated from different regions of the body, such as the area of the tonsils, carious lesions, salivary stones, periodontal pockets, mucosa of the oropharynx, and gastrointestinal and urogenital tracts [4, 5].

*Actinomycetes* cause chronic, slow-growing infections, especially when normal mucosal barriers have been disrupted by trauma, surgery, or previous infection [1, 4, 5]. Actinomycosis is usually a polymicrobial infection with the presence of associated strains of bacteria, most commonly streptococci, spindle-shaped or Gram-negative bacilli, and *Haemophilus* species [2–4, 6]. The associated flora forms a kind of symbiosis with *Actinomycetes* species (*Actinomyces viscosus*, *Actinomyces naeslundii*, and *Actinomyces odontolyticus*) and can cause an anaerobic environment that supports the growth of this type of microorganisms [6–8]. These associated bacterial species function as copathogens, contributing to infection development by producing toxins and enzymes, which inhibit the host defenses [5, 6]. These companion species enhance the relatively low invasiveness of *Actinomycetes* by causing early manifestations of infection and failure to conduct treatment. *Actinomycetes* do not contain sterols in their cell walls and are sensitive to antibacterial chemotherapeutic agents [6, 8–10].

This infection is anatomically and clinically divided into three types: cervicofacial, pulmonary, and abdominal pelvic, the first being the most common form [2]. Bone involvement is rare, with osteomyelitis occurring sporadically, secondary to existing portal injuries caused by tooth extraction, trauma to the jaw, diabetes, immunosuppression, long-term corticosteroid treatment, alcoholism, and smoking [4, 5, 7]. The infection progresses by direct or hematogenous spread to surrounding tissues. Actinomycosis affects the tissues not following the usual anatomical planes, but rather breaking through them and becoming a lobular “pseudotumor” [6, 7].

In 55% of cases, there may also be involvement of the eyes and various other systems including nervous, respiratory, urogenital, and digestive. The most commonly affected age group ranges from 30 to 60 years, with a male to female ratio of 4:1. Clinically, the infection presents as an acute or chronic form, with the former being significantly less common [5, 6, 8–10].

This case report is aimed at presenting a rare case of actinomycotic osteomyelitis in the lower jaw of a patient who is not immunocompromised or has any comorbidities.

## 2. Case Presentation

A 29-year-old man with no underlying health conditions was referred to the Faculty of Dental Medicine at the Medical University of Sofia, Department of Dental, Oral, and Maxillofacial Surgery, due to intermittent suppuration from the lower jaw in the area of Tooth 47, which had been extracted approximately 5 years prior.

The patient has undergone multiple courses of oral antibiotics, including amoxicillin, clindamycin, and azithromycin over this period. However, despite these treatments, the condition has not improved.

Microbiological evaluations were not performed during follow-up consultations.

**Figure 1:**
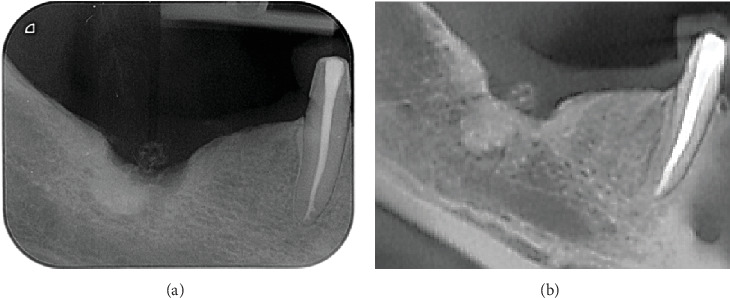
(a) Preoperative intraoral periapical radiograph of the region of missing mandibular right molars and (b) a reconstructed panoramic CBCT image (cropped) through the same region, revealing a bony sequestrum, slight soft tissue swelling, and sclerotic area in the underlying bone.

Clinical examination revealed the presence of a fistula/sinus tract intraorally in the region of the extracted Tooth 47 without redness of the overlying mucous membrane. Upon applying slight pressure to the gingiva in the area, a purulent exudate was noted. The site exhibited mild tenderness, and on palpation, no signs of lymphadenopathy were present. A segmental radiograph of the affected area showed ill-defined bony changes with osteolytic and osteosclerotic areas (Figures 1(a) and 1(b)).

Based on the patient's history, clinical presentation, and radiographic findings, a preliminary diagnosis of chronic osteomyelitis of the mandible was made.

**Figure 2:**
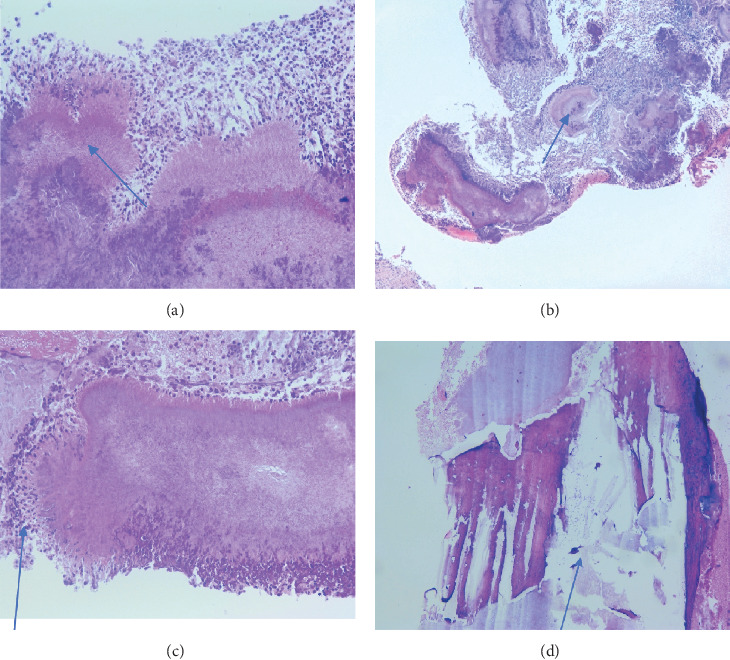
(a, b) Histological features of Splendore–Hoeppli phenomenon appearing as radiating eosinophilic material around bacterial colonies due to deposition of antigen–antibody complexes and debris from the inflammatory cells. Basophilic bacterial aggregates with filamentous periphery surrounded by neutrophils and chronic granulomatous inflammation (H&E stain, ×40). (c) Large basophilic bacterial aggregates (sulfur granules) surrounded by neutrophils (H&E stain, ×5). (d) Fragments of necrotic bone (H&E stain, ×5).

Surgical intervention was proposed to the patient. A triangular mucoperiosteal flap was performed under local anesthesia with a mandibular block. The affected area was exposed, and careful curettage was performed. The pathologically altered tissue was removed, which revealed yellowish granules, clinically indicative of actinomycotic colonies. These granules, known as “sulfur granules,” are characteristic of actinomycosis. A peripheral ostectomy was performed with a round bur until the well-vascularized bone was visualized. After smoothing and adequate hemostasis, the wound was sutured. The tissues and sequestrum removed during the surgical procedure were submitted for histopathological evaluation. On histopathological examination (No. Ti455/28.07.2023), trabeculae of necrotic braided bone and *Actinomycetes* granules with bone marrow and multiple partially resorbed bone sequestrum were observed. Nonspecific inflammatory cells, vascular proliferations, and granulation tissue were also observed. Within the granulation, tissue granules were surrounded by polymorphonuclear cells (Figures 2(a), 2(b), and 2(c)). The periphery of *Actinomycetes* granules showed radiating, basophilic filaments and eosinophilic, club-shaped ends.

Actinomycosis is an indolent pyogranulomatous disease that can cause destruction of the underlying bone (Figures 2(b) and 2(d)). The exact pathologic diagnosis requires the constellation of dense filamentous bacterial aggregates and associated infiltrate of neutrophils and granulation tissue (Figures 2(b), 2(c), and 2(d)).

Therapy with oral Augmentin 2 × 1.0 g for 10 days was prescribed. The postoperative period was without any complications, and antibiotic treatment was continued with amoxycillin 2 × 1.0 g for a period of 1 month.

**Figure 3:**
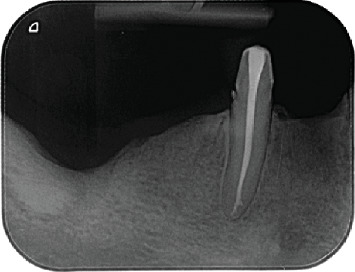
Intraoral periapical radiograph of the region 5 months postoperatively.

The patient was followed up for 5 months, with no evidence of disease recurrence (Figure 3).

## 3. Discussion

Actinomycosis is an uncommon saprophytic infection characterized by granulomatous lesions caused by resident microorganisms in the oral cavity and oropharynx including *Actinomycetes*—*A. israelii*, *A. viscosus*, *A. odontolyticus*, *A. naeslundii*, and *A. meyeri*, and is not common in clinical practice [9]. It manifests as acute, subacute, or chronic form and involves only soft tissue, bone (osteomyelitis), or both [8]. There is a 4:1 ratio for predilection in females. In our case, it refers to a male patient post extraction of a tooth. The lower jaw was involved with the presence of an intraoral fistula with periodic suppuration as described in other studies [8, 11, 12]. Similar colonies (*A. israelii*, *A. viscosus*, *A. odontolyticus*, *A. naeslundii*, and *A. meyeri*) can also be isolated in drug-induced osteonecrosis of the jaw bones [13]. These organisms have a low virulence potential, but the resident bacterial flora act as copathogens and lead to infection by releasing an enzyme and a toxin. This polymicrobial oral microbiome works synergistically as a specific ecosystem with reduced redox potential which is favourable for anaerobic growth. As a result, the highly vascularized tissue is replaced by a poorly vascularized granulation tissue, ensuring anaerobic environment [14]. This may explain the prolonged progression of the infection, which, in our case, persisted for nearly 5 years. Although the pathogenesis of actinomycotic osteomyelitis is not completely understood, it is assumed that inflammation starts when the normal oral microbiota is unbalanced and localized pathological changes in the jaw bone could be detected due to chronic inflammation and cause sclerotic changes, as in the case we described.

Numerous studies suggest that the mandibular location of the disease may result from the relatively poor vascularization of the lower jaw [4, 5, 7, 10, 12, 15].

In the present case, the infection was manifested as a localized granulomatous inflammatory process with central purulent necrosis of the mandible in the area of an extracted 47, consisting of granulation tissue with aggregates of bacterial filamentous colonies with associated suppuration from the intraoral sinus tract. This is typical for actinomycosis as it is described in other reports [4, 5, 7, 10], but sometimes, the process of granulomatous inflammation leads to the formation of bone spicules rather than complete lytic bone destruction and can mimic other diseases [10, 16, 17].

Radiographs in our case showed endosteal sclerosis, in line with findings from other reported cases [5, 8, 13], likely due to repeated cycles of symptom exacerbation and remission throughout multiple antibiotic treatments.

It can cause a local sclerosing type of osteomyelitis, which may mimic bone tumours [10, 17] or a bone substitute material or residual root [14, 15]. Delay of several years in establishing an accurate diagnosis was described in different reports [4, 8, 14, 15]; in the present case, we describe 5 years period of misdiagnosis. While patient history and physical examination are fundamental in diagnosing diseases, actinomycotic osteomyelitis requires additional diagnostic approaches. These include imaging studies, bacterial cultures, and microscopic analysis of tissue samples from the infection site [8, 10, 12]. In our clinical case, there was no preoperative-specific clinical data for actinomycosis other than the presence of only an intraoral fistulous course with periodic discharge of pus. Regional lymphadenopathy was absent, which is similar to other studies [8, 14–16]. Radiographs can be useful in recognizing the spread of the infectious process in the bones but are nonspecific for actinomycosis [12, 14]. MRI, CT, and gallium scintigraphy can be used to differentiate inflammatory changes from neoplasm, and CT scan can be performed to establish osteolysis and formation of fibrous tissue in the infected area [14]. Scintigraphy can be useful to determine the effectiveness of therapy. However, no single imaging modality can be used as the sole form of diagnosis [8]. In our case, an osteolytic focus with osteosclerosis was found peripherally on cone beam computed tomography (CBCT), which resembled a bone graft/root similar to radiological changes described in other articles [14–16].

Ancillary microbiological tests, for example, bacterial culture, are a definitive method for diagnosing actinomycosis; however, in this case, it was not performed. Isolation and identification of *Actinomyces* can be challenging due to fastidious nature and slow growth rate, as well as prior antibiotic therapy, overgrowth of contaminant microorganisms, and inadequate culture conditions or incubation periods [1, 9, 11, 17, 18]. If the culture is poorly performed, delayed, or the patient has had concomitant or recent antibiotic therapy, the result may be negative [19]. PCR technique might be more efficient in the diagnosis of actinomycosis in comparison to culture and smear microscopy. Microscopic diagnosis is indicative in the presence of sulfur granules. Exfoliative cytology allows the identification of sulfur granules produced by *Actinomyces*, providing a rapid diagnosis of actinomycosis. Cytological examination also reveals *Actinomyces* surrounded by a large number of neutrophile granulocytes [11, 13, 18].

As reported in other articles, the histological finding of our case is typical and indicative of actinomycosis [4, 5, 11–13]. Gram staining reveals Gram-positive interlacing branching filaments along with Gram-positive and negative cocci and bacilli [11]. The filaments are negative for acid-fast and positive for PAS (periodic acid–Schiff stain) [11, 15]. Many authors state that in actinomycosis, biopsy of the soft tissue and adjacent exposed necrotic bone revealed stratified squamous epithelium with naked basophilic bacterial colonies resembling actinomycotic colonies [4, 11, 15, 16]. Necrotic bone shows sclerosis with actinomycotic colonies in bone sequestrations and on the trabecular surface [13, 15].

Treatment of actinomycotic osteomyelitis consists of comprehensive surgical treatment—curettage, sequestrectomy, and peripheral osteotomy [2, 12]. Medication includes administration of high doses of penicillin, depending on the severity of the case. It can be prescribed as intravenous infusion or oral administration of 2–4 g per day over a period of several months, depending on the severity of the infection and presence of systemic symptoms [4, 9, 15]. Following the radical surgical intervention and considering the patient's favourable general condition and positive response to treatment, we administered a beta-lactam antibiotic regimen of amoxicillin at a dosage of 1.0 g twice daily for 1 month.

Other antibiotics that are effective include clindamycin, erythromycin, chloramphenicol, cephaloridine, minocycline, and imipenem [5, 12]. Metronidazole and aminoglycosides are ineffective against *A. israelii* [11, 12]. Some studies revealed that monoinfection with *Actinomycetes* causes acute purulent infections, though mixed infection with *Eikenella* infections and *A. actinomycetemcomitans* cause chronic, persistent inflammation and form the basis for recommending high doses of antibiotics [17, 18, 20]. Early diagnosis and timely therapy can significantly improve the outcome of the disease [8, 9, 16, 20].

Untreated cervicofacial actinomycosis can disseminate to distant organs, including the lungs, brain, and digestive tract. This condition does not show a preference for age, race, season, or occupation. However, certain risk factors increase the susceptibility, such as male gender, poorly controlled diabetes, immunosuppression, alcoholism, and malnutrition.

Actinomycosis can closely resemble malignancies, making accurate diagnosis challenging. To differentiate between these conditions, various diagnostic tools are essential such as CT, MRI, scintigraphy, biopsy, and PCR to establish a definitive diagnosis.

## 4. Conclusion

*Actinomycetes* can cause deep soft tissue and/or bone infections when there is a break in the mucosal barrier or under favourable conditions, typically with a chronic clinical course. Microbiological cultures may not always yield positive results; however, histopathology can often assist with definitive evidence for a diagnosis. Actinomycosis is a completely curable disease, and with timely diagnosis and adequate therapy (surgical and medication), functional and aesthetic impairments can be minimized.

## Data Availability

The data used to support the findings of this study are included within the article.
